# Clinical and epidemiological study of onychomycosis among patients of the national institute of hygiene in rabat, Morocco (2016-2020)

**DOI:** 10.22034/CMM.2024.345174.1492

**Published:** 2023-12

**Authors:** Majda El Abbassi, Boughroud Hajar, Fatima Amarir, Rhajaoui Mohamed, Adlaoui El Bachir, Mkinsi Hanane, Baha Imane, Rais Samira

**Affiliations:** 1 National Institute of Hygiene, Agdal-Rabat, Morocco; 2 Immunology and Biodiversity Laboratory, Faculty of Sciences Ain Chock, Hassan II University, Casablanca, Morocco; 3 Department of Biology, Faculty of Science Ben M’Sick, Hassan II University, Casablanca, Morocco

**Keywords:** Dermatophytes, Fungal infection, Mixed infection, Nail mycosis, Yeasts

## Abstract

**Background and Purpose:**

Onychomycosis is a common nail infection characterized by the discoloration, thickening, and detachment of nails. This study aimed to provide valuable insights into this pathology by assessing its prevalence, clinical aspects, related comorbidities, and causative agents in patients from a Moroccan population.

**Materials and Methods:**

This retrospective study was conducted on 1,606 subjects at the Mycology-Parasitology laboratory of the National Institute of Hygiene in Rabat, Morocco, over five years (2016-2020). Nail samples were collected from both fingernails and toenails and processed through microscopic examination and culture. The incubated tubes were kept at a temperature range of 28-30°C for 4-5 weeks.

**Results:**

Onychomycosis was mycologically confirmed in 1,794 samples (93.24%). It occurred commonly in the 41-60 age group, with a higher incidence among females (74.53%). Diabetes, alongside other chronic diseases, was prevalent among patients with underlying conditions, comprising 131 cases (40.56%). Disto-lateral subungual onychomycosis emerged as the most prevalent clinical presentation, comprising 1,536 cases (79.92%). Fingernails primarily affected by yeasts, notably *Candida albicans*, accounted for 565 cases (29.80%), while toenails were predominantly impacted by dermatophytes, primarily *Trichophyton rubrum* (n=1,230, 64.87%). Mixed infections exclusively featured yeasts and dermatophytes, predominantly *T. rubrum* and *C. albicans*, which accounted for 79 (4.40%) cases. The study explored the influence of molds, yielding insights into their rarity in onychomycosis.

**Conclusion:**

These findings hold significant implications for the clinical management and diagnosis of onychomycosis, particularly in patients with underlying chronic conditions. Further epidemiological studies across Morocco are needed for meaningful comparisons.

## Introduction

Onychomycosis, a fungal nail infection, is characterized by nail thickening, detachment from the nail bed, and discoloration [ 1 ]. It stands as the most common nail infective disorder, accounting for approximately half of all consultations related to nail disorders [ 2 ]. The disease is often influenced by humid environments, tight footwear, and genetic predisposition [ 3 ]. Although it can manifest at any age, its occurrence notably rises in the elderly population [ 4 ]. Concurrent health conditions, including cancer, diabetes, immune deficiency, or peripheral arterial disease, can increase vulnerability to developing onychomycosis [ 5 ].

Its classification relies on the assessment of the clinical presentation and the route of invasion, resulting in four primary types, namely distal subungual onychomycosis, proximal subungual onychomycosis or proximal white subungual onychomycosis, white superficial onychomycosis (WSO), and total dystrophic onychomycosis (TDO) [ 4 ].

 Diagnosis involves reviewing the medical history of the patient, conducting a physical examination, and utilizing microscopy and culture techniques to analyze nail specimens [ 6 ]. Fungal dermatophytes, specifically anthropophilic strains, such as *Trichophyton rubrum* and *Trichophyton interdigitale*, commonly serve as the etiological agents responsible for onychomycosis [ 2 ]. Molds other than dermatophytes are a less frequent cause within the general population, whereas *Candida* species are a prevalent cause of yeast onychomycosis [ 3 ].

Despite its perception as primarily a cosmetic issue, a study performed by Aditya K. Gupta and Rachel R. Mays in 2017 affirmed the substantial physical and psychological impact onychomycosis can have on patients [ 1 , 7 ]. The selected treatment varies based on its type, severity, and the existing medical conditions of the patient. However, the treatment necessitates a minimum of three months, given the slow growth rate of nails [ 2 , 8 ]. Due to the duration and associated costs, patients often struggle to adhere to the therapy [ 6 ].

In Morocco, the prevalence of onychomycosis is possibly underestimated due to the prohibitive costs of treatment, which hinder access to mycological examinations. Moreover, comprehensive epidemio-logical studies on a large scale have yet to be conducted to provide an accurate assessment [ 9 ]. Prior research conducted in Casablanca and Marrakech in Morocco has reported high prevalence rates, ranging between 64.5% and 72.2% [ 9 , 10 ]. In this regard, the present study aims to assess the prevalence, clinical manifestations, associated comorbidities, and causative agents of onychomycosis using specimens obtained over five years (2016-2020) and diagnosed at the Mycology-Parasitology laboratory of the National Institute of Hygiene in Rabat.

## Materials and Methods

This study was conducted over five years, from January 4, 2016, to December 23, 2020, on samples of 1,606 patients with clinically suspected onychomycosis. Patients underwent detailed medical history assessments, encompassing inquiries about their use of topical or systemic antifungal medications. A structured data sheet was utilized to record the name, age, and gender of patients, as well as the site of infection, underlying conditions, and clinical presentation. Microbiological analyses were performed on collected samples, including fingernail and toenail clippings from lesions clinically suspected of fungal infections, as well as scraping from intertriginous or hyperkeratotic lesions.

Prior to specimen collection, the affected area was cleansed with 70% ethanol to prevent contamination. Using sterile nail clippers, clippings were obtained by scraping the nail bed, the underside of the nail plate, and the hyponychium. The collected specimens underwent an analytical process, starting with a direct microscopy and culture assessment. Initially, the specimens were exposed to a 20% potassium hydroxide (KOH) solution to facilitate the visualization of mycelial filaments, pseudomycelial filaments, or budding yeasts.

Subsequently, nail specimens were inoculated onto three culture media, comprising one with Sabouraud Dextrose Agar (SDA, Oxoid, UK), one duplicate in SDA supplemented with chloramphenicol (0.05 µg/ml, Oxoid, UK), and one SDA supplemented with both chloramphenicol and cycloheximide (0.5 µg/ml, Oxoid, UK). The inoculated SDA tubes were incubated within a temperature range of 28-30°C for 4-5 weeks. The tubes were examined thrice weekly for any signs of fungal growth, with no growth observed by the fourth week being considered a negative culture.

Identification was based on a set of criteria, such as growth rate, especially focusing on the macroscopic and microscopic aspects of primary culture colonies. Dermatophytes were evaluated for color, colony shape, texture, consistency, and surface characteristics on both sides of the culture media. The reverse side of the culture was inspected for deep agar formations, with any pigment diffusion being noted. Further identification was performed using the Roth's Flag technique under microscopic examination. Additionally, the urease test aided in distinguishing between *T. interdigitale* and *T. rubrum*.

The clinical validation of non-dermatophytic molds (NDMs) pathogenicity necessitates positive samples, particularly when isolated without co-existing dermatophytes. This determination relied on the colony morphology and microscopic examination of lactophenol cotton blue preparations. Yeasts were identified based on morphology, and the confirmation of Candida spp. involved observing pseudomycelium and blastospores under light microscopy, conducting a germ tube test, and using the AUXACOLOR 2 (BIO-RAD, France) rapid identification system.

The descriptive data were transcribed onto a data sheet and entered into Microsoft Office Excel software (version 2010) for subsequent analysis (https://support.microsoft.com). These data were analyzed by Epi Info™ software (version 7.2.5) for Windows (https://epi-info.software.informer.com/7.2/). The Chi-squared test (χ2) was used, and a P-value of <0.05 was considered statistically significant. When the Chi-squared test was not valid, Fisher's exact test was applied. Moreover, graphs were generated using GraphPad Prism (version 8.0.2.) (https://www.graphpad.com).

## Results

### Study Population and Period

Between January 2016 and December 2020, the laboratory received a total of 2,010 individuals with various dermatological presentations, including onychopathies, palmar and plantar keratodermas, and ringworm, as well as inflammatory skin lesions and axillary folds. The attendance at the laboratory experienced significant fluctuations due to the impact of the COVID-19 pandemic. Notably, the number of patients decreased from 427 in 2018 to 160 in 2020.

In total, 2,445 samples were collected from the screened individuals, as multiple samples per patient were required for infections that affected multiple anatomical sites. Within this sample pool, 1,922 (78.60%) specimens were collected from 1,606 patients presenting clinical signs of possible onychomycosis. It was noticed that the prevalence was higher in females (n=1,197, 74.53%) than males (n=409, 25.47%). The average age of the participants in the study was 47.88±17.42 years. However, age data were not available for 11 individuals (0.68%). Fungal infections were most common among patients within the age range of 41-60 (n=734, 45.7%), followed by those over 60 (n=381, 23.73%). Patients aged 21-40 and under 20 accounted for 21.8% (n=350) and 8.1% (n=130) of the studied population, respectively (Table 1).

**Table 1:** Prevalence of onychomycosis based on age and gender

		Total number of patients examined (Male/Female	Percentage (%)
**Age groups (years)**	0-10	57 (28/29)	3.55
	11-20	73 (19/54)	4.55
	21-30	126 (17/109)	7.85
	31-40	224 (47/177)	13.95
	41-50	353 (75/278)	21.98
	51-60	381 (95/286)	23.72
	61-70	245 (73/172)	15.26
	71-80	117 (42/75)	7.29
	>80	19 (10/9)	1.18
	Without age	11 (3/8)	0.68
Total		1606 (409/1197)	100
	Chi-squared χ2	48.608	
	P-value	0.001*	

* A high statistically significant difference (P<0.001)

### Underlying Conditions

In this study, 254 (15.82%) out of the 1,606 patients under investigation reported underlying comorbidities, with some presenting up to four concurrent clinical conditions. In total, 275 chronic diseases were reported, with diabetes identified as the most prevalent, accounting for 40.56% of the cases (n=131). Autoimmune diseases were evident in 23 patients (7.12%), while an additional 20 patients (6.19%) exhibited diverse pathologies, such as anemia. Infectious diseases were less common, reported in only 5 patients (1.55%). Table 2 provides further insight into the underlying conditions observed.

**Table 2:** Detailed underlying conditions in each category

Categories	Underlying conditions	Patients
Chronic diseases	Diabetes	131
	Hypertension	98
	Heart diseases	19
	Hypercholesterolemia	13
	Asthma	6
	Cancer	3
	Depression	2
	Neuropathy	1
	Osteoporosis	1
	Renal failure	1
Autoimmune diseases	Rheumatism	9
	Goiter	6
	Allergy	3
	Lupus erythematosus	2
	Behcet	1
	Psoriasis	1
	Skin hypersensitivity	1
Other	Anemia	16
	Trisomy 21	1
	Prostate disease	1
	Glaucoma	1
	Sciatiques	1
Infection diseases	Hepatitis C	2
	Zona virus	1
	HIV	1
	Erysipelas	1
Total		323

### Clinical Presentation

The prevalent clinical presentation was Distolateral Subungual Onychomycosis (DLSO), observed in 1,536 (79.92%) of the affected nails. The association between onyxis and perionyxis was rare, observed in only 5 (0.26%) cases. The WSO and TDO accounted for 9 (0.47%) and 2 (0.10%) cases, respectively. Within DLSO, three significant associations were noted, mainly with paronychia (n=251, 13.06%), followed by palmoplantar keratosis (n=73, 3.80%) and intertrigo (n=46, 2.39%), as outlined in Table 3.

**Table 3:** Clinical presentations in the infected sites

		Nail site (n)		Total (%)
		Fingernail	Toenail	
**Clinical presentations**	DLSO	532	1004	1536 (79.92)
	DLSO/Paronychia	74	177	251 (13.06)
	DLSO/Palmoplantar keratosis	20	53	73 (3.80)
	DLSO/Intertrigo	11	35	46 (2.39)
	Onyxis/Perionyxis	3	2	5 (0.26)
	WSO	5	4	9 (0.47)
	TDO	1	1	2 (0.10)
Total (%)		646 (33.61)	1276 (66.39)	1922 (100)
	Fisher’s exact	0.1049			
	P-value	0.1432			

A statistically significant difference (P<0.05)

DLSO: Distolateral subungual onychomycosis, WSO: White superficial onychomycosis, TDO: Total dystrophic onychomycosis

### Microscopic and Culture Examination

As illustrated in Table 4, a total of 1,922 samples were identified as possibly suspected cases of onychomycosis through mycologic examinations. Among these, a majority of 1,792 samples (93.24%) resulted positive on both culture and direct microscopic examinations (DME). Two samples (0.10%) were positive only in culture, nine (0.47%) were only positive in DME, and 119 samples (6.19%) tested negative on both culture and DME.

**Table 4:** Comparison and distribution of potassium hydroxide and culture findings

KOH and culture observation	Number of samples (M/F)	Percentage (%)
KOH Positive, Culture Positive	1792 (473/1319)	93.24
KOH Positive, Culture Negative	9 (0/9)	0.47
KOH Negative, Culture Positive	2 (0/2)	0.10
KOH Negative, Culture Negative	119 (27/92)	6.19
Total	1922 (500/1422)	100
Fisher’s exact	0.231	
P-value	0.196	

A statistically significant difference (P<0.05)

KOH: Potassium hydroxide

### Mycologic Database

**Figure 1:**
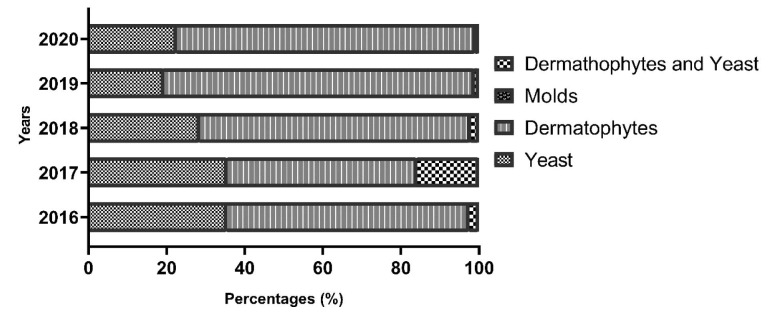
Pathogen group distribution over time (2016-2020)

The graph in Figure 1 illustrates the distribution of identified pathogen groups over the study period. Dermatophytes have consistently emerged as the predominant pathogen group causing onychomycosis. Remarkably, yeasts held the second-ranking position. The coexistence of dermatophytes and yeasts was observed in all years of the study. In contrast, molds were ranked the lowest, with only one recorded case in 2018.

The analysis of positive cases in terms of gender revealed variations regarding the anatomic site and the fungal causative agent. Results indicated a higher prevalence of infections in fingernails (n=425, 23.69%) and toenails (n=896, 49.94%) among females, compared to males. The causative agents in fingernails and toenails were identified in 169 (9.42%) and 304 (16.95%) males, respectively, which was notably lower than that in females.

The findings of the present study showed that dermatophytes were present in 1,251 (65.98%) positive cultures. Moreover, *T. rubrum* was the most frequently isolated pathogen, which was found in 1,230 (64.87%) cases, followed by *T. interdigitale* accounting for 21 (1.11%) cases. In toenail onychomycosis, dermatophytes were the prevalent pathogens, with 977 (84.96%) positive cultures; however, fingernail onychomycosis was found in 173 cases (15.04%).

Data also revealed that yeasts were isolated in 644 (33.97%) positive cultures, with a higher frequency in fingernails (n=396, 73.06%), compared to toenails (n=146, 26.94%). The most frequently isolated fungus was *C. albicans* with 565 samples (29.80%), followed by *C. parapsilosis* with 51 samples (2.69%) and *Trichosporon* spp. with 28 samples (1.48%).

Regarding molds, only one case (0.05%) was registered in the toenail of a female, where *Aspergillus* spp. was identified as the causative agent. Among mixed infections, which accounted for only 101 (5.63%) cases, the most common combination was *T. rubrum* and *C. albicans*, found in 79 (4.40%) cases. Notably, toenails exhibited the highest prevalence, with 76 (75.25%) reported cases. One exceptional instance (0.06%) worth noting involved the isolation of three causative agents from the toenail of a female, namely *T. rubrum*, *C. albicans*, and *Trichosporon* spp. Table 5 provides additional details about the findings.

**Table 5:** Distribution sites and causative agents in confirmed onychomycosis cases

Fungal agents	Fungal species	Fingernail		Toenail		Number (%)
		Male	Female	Male	Female	
**Yeast**	*Candida albicans*	76	287	21	101	485 (27.03)
	*Candida parapsilosis*	9	21	-	12	42 (2.34)
	*Trichosporon* spp.	1	2	1	11	15 (0.84)
Dermatophytes	*Trichophyton rubrum*	64	105	265	695	1129 (62.93)
	*Trichophyton interdigital*	1	3	2	15	21 (1.17)
**Molds**	*Aspergillus* spp.	-	-	-	1	1 (0.06)
**Dermatophytes and Yeasts**	*T. rubrum*+*C. albicans*	15	7	10	47	79 (4.40)
	*T. rubrum*+*C. parapsilosis*	2	-	2	4	8 (0.45)
	*T. rubrum*+*Trichosporon*	1	-	3	9	13 (0.72)
	*T. rubrum*+*C. albicans*+*Trichosporon*	-	-	-	1	1 (0.06)
**Total**		169 (9.42)	425 (23.69)	304 (16.94)	896 (49.94)	1794 (100)
	Fisher’s Exact	0.001*			0.0091*	
	P-value	0.001*			0.0466*	

* A statistically significant difference (P<0.001)

## Discussion

With a global range of 18-50%, the prevalence of onychomycosis is on the rise, constituting 2-18% of dermatology consultations [ 6 , 9 , 11 ]. In Morocco, onychomycosis is significantly common, with a diverse range of fungi contributing to this infection [ 12 ]. Over a five-year period, a total of 1,606 patients visited the Mycology-Parasitology laboratory of the National Institute of Hygiene in Rabat, seeking consultation for suspected fungal nail infections.

The attendance in the laboratory underwent substantial changes as a result of the COVID-19 pandemic. Many healthcare systems were forced to allocate resources to manage the influx of COVID-19 patients. Moreover, lockdown measures were implemented to minimize exposure to the virus, thereby limiting contact with potential sources of contagion in public places, such as swimming pools, steam baths, mosques, and gyms. These measures might have possibly contributed to the decrease in patient consultations during this period.

Nail fungus has been identified as a disease exhibiting gender- and age-related variations [ 2 ]. The present study revealed a higher prevalence in females (74.53%) than in males (25.47%). Other researchers have also reported this predominance [ 13 - 15 ]. This could be attributed to the tendency of females to prioritize higher cosmetic standards, compared to males, coupled with their frequent involvement in household chores, resulting in increased exposure to wet and chemical-laden cleaning products. Conversely, other studies have reported an opposite trend [ 16 , 17 ].

A high onychomycosis prevalence was observed within the 41-60 age group, in line with previous studies [ 15 , 17 - 19 ]. This reinforces the age-related manifestation of onychomycosis, typically occurring between the fourth and sixth decades of life, which can be associated with factors such as frequent minor injuries and increased physical activity among this age category [ 9 , 17 ].

A high incidence of onychomycosis, attributed to various factors, including underlying medical conditions, presents a significant health risk when it co-occurs with other organic diseases due to potential drug interactions during oral therapy [ 20 , 21 ]. In the present study, only a minority of 15.75% had coexisting diseases, with diabetes being the most common comorbidity. These findings are consistent with those of prior studies conducted in eastern Nepal and Mexico, emphasizing diabetes as a significant comorbidity in onychomycosis patients [ 5 , 16 , 21 ]. Notably, nearly one-third of diabetic patients experience onychomycosis [ 5 ]. This is explained by decreased foot sensation and increased vulnerability to trauma, which damages nails and creates routes for fungal infections. Furthermore, diabetes-related neuropathy and vascular insufficiency contribute to nail bed injuries, further predisposing individuals to fungal invasions [ 20 ].

The present study identified DLSO as the most prevalent clinical presentation (79.92%), which is in line with the findings of other studies [ 15 , 18 , 22 ]. It was also noted that there were associations between DLSO and paronychia (13.06%), palmoplantar keratosis (3.80%), and intertrigo (2.39%). These figures were slightly lower than previously reported rates [ 11 , 18 , 23 , 24 ]. In contrast, this study reported a notably lower incidence of perionyxis (0.26%), while Sylla et al. found a higher rate of 10% [ 24 ].

Additionally, WSO and TDO were relatively uncommon, which is consistent with the findings of a previous study conducted in Egypt [ 19 ]. These findings support the notion that DLSO is the primary clinical manifestation of onychomycosis. Its high prevalence rate may be attributed to its progressive nature, which facilitates the concealment of the infection until its advanced stages. In addition, underlying foot infections, such as athlete's foot, are often associated with its occurrence, providing an optimal environment for the fungal invasion of the nail bed and underside of the nail plate [ 4 , 25 ].

Diverse diagnostic methods, including KOH testing, culture, histopathology, dermoscopy, and advanced techniques, such as molecular assays and artificial intelligence, assist in diagnosing onychomycosis [ 8 ].

Based on the findings, 1,506 out of 1,606 patients tested positive either on culture, DME, or both, accounting for a prevalence of 93.77% within the cohort. It is noteworthy that the present investigation showed a notably high positivity rate of 93.24% in both microscopy and culture, contrasting with Ilkit's rate of 33.5% [ 26 ]. On the one hand, culture alone yielded only a 0.10% positivity rate, while DME produced a 0.47% rate, both of which were notably lower than the rates documented by Gregoriou et al. [ 14 ]. On the other hand, 6.19% of the cases yielded negative results in both microscopy and culture, less than that in a study performed in Northeast Brazil [ 17 ].

These variations are due to differences in laboratory methods for the analysis of onychomycosis samples, notably in sampling, culture techniques, and microscopic examinations. Additionally, factors such as the infrequent repetition of mycological examinations or the presence of lesions deemed too old for accurate detection contribute to these discrepancies [ 27 ]. The expertise of the laboratory staff also plays a significant role in the accuracy of the results [ 16 ].

Dermatophytes were the predominant group of fungi, accounting for 65.98% of the samples. This is consistent with the results of studies conducted in both Morocco and Cameroon, showing similar isolation rates of 65% and 66.6%, respectively [ 9 , 22 ]. In contrast, studies carried out in Senegal and Greece found a higher prevalence rate of yeasts, while research in Pakistan showed no statistically significant difference between yeast and dermatophyte infections [ 14 , 15 , 24 ]. *T. rubrum*, the most commonly isolated organism in global nail fungus cases, was also the predominant species in the present study, with a prevalence rate of 65.98%, followed by *T. interdigitale* (1.11%). These findings are in line with observations made by Godoy-Martinez et al. [ 11 , 28 ]. The high attendance in moist environments, such as Moroccan bathhouses and ablution areas, increases the risk of fungal infections of this anthropophilic species among the Moroccan population [ 9 ].

*Candida* spp. emerge as secondary pathogens, with *C. albicans* declared the most important yeast-like pathogen [ 29 ]. The data collected in this study supported this characterization, as yeasts were the second most common manifestation, consistent with a prior study [ 22 ]. *C. albicans* (33.97%) emerged as the primary isolated causative agent, followed by *C. parapsilosis* (2.69%) and *Trichosporon* spp. (1.44%). This pattern is in line with the findings of a study conducted in northeastern Brazil [ 17 ].

Regarding molds, only one case of *Aspergillus* spp. was identified in the toenail of a female (0.05%). According to Hajoui et al., the prevalence of opportunistic NDMs reached 2.78%, confirming their generally low occurrence worldwide [ 2 , 30 ]. Diagnostic complexity arises from common mold contamination in nail samples and mycology laboratories, often resulting in false-positive results. The lack of repetitive culture, along with adequate mycological methods for mold cultivation, also helps to explain this rarity [ 30 , 31 ].

Etiological agents may manifest individually or as part of a mixed infection [ 32 ]. The findings of the present study indicated their relative rarity, accounting for 5.63% of the cases, a pattern consistent with the results of a study performed in Hong Kong [ 33 ]. These mixed infections exclusively featured combinations of yeasts and dermatophytes, with the most common pair being *T. rubrum* and *C. albicans*, which accounted for 78.22% of the cases. This is consistent with the findings of a study carried out in Malaysia [ 34 ]. However, Gupta and Nakrieko, using molecular analysis, reported a higher rate of mixed infections, with 41% of patients testing positive, primarily involving dermatophytes and NDMs [ 35 ].

Regarding the site of infection, a higher prevalence of onychomycosis was observed in toenails (66.88%), compared to fingernails (33.11%). These findings are consistent with those of a retrospective study performed on 2,070 nail samples, 75% and 23% of which were from toenails and fingernails, respectively [ 9 ]. This can be attributed to the greater pressure endured by toenails due to the weight of the body and the confined nature of footwear, making this condition 25 times more likely to occur in toenails than in fingernails [ 25 ].

Moreover, yeasts predominantly affected fingernails (73.06%), while dermatophytes were more commonly found in toenails (84.96%). Fingernails, primarily affected by yeasts, notably *C. albicans*, accounted for 574 cases (31.99%), while toenails were predominantly impacted by dermatophytes, primarily *T. rubrum*, with 1,114 cases (62.1%). The different location preferences of these causative agents could be attributed to variations in the nail microenvironment, particularly in terms of temperature, humidity, and pH [ 1 ].

## Conclusion

The present study disclosed that the incidence of onychomycosis cases was impacted by the COVID-19 pandemic. In Rabat, a significant proportion of onychomycosis cases were observed in individuals within the age range of 41-60. A noteworthy association between onychomycosis and chronic diseases, particularly diabetes, was observed among patients with underlying conditions. Moreover, DLSO emerged as the prevalent clinical manifestation.Among the research samples of the present study, molds and mixed infections were infrequent, while dermatophytes were the most commonly isolated pathogen group, particularly in toenails. Conversely, yeasts were more prevalent in fingernails. The predominant species identified were *T. rubrum* and *C. albicans*. Further studies on the epidemiological characteristics of onychomycosis across Morocco are needed to enable meaningful comparisons.
